# Utilization of MRI in surgical decision making in the shoulder

**DOI:** 10.1186/s12891-022-05541-0

**Published:** 2022-06-18

**Authors:** Maciej J. K. Simon, William D. Regan

**Affiliations:** 1 Department of Orthopaedics and Trauma Surgery, University Medical Center Schleswig-Holstein, Campus Kiel, Arnold-Heller-Strasse 3, 24105 Kiel, Germany; 2 Department of Orthopaedics, University of British Columbia, Chan Gunn Pavilion, Allen McGavin Sports Medicine Clinic, 2553 Wesbrook Mall, Vancouver, BC V6T1Z3 Canada

**Keywords:** MRI scans, MRI reports, Shoulder pathology, Surgical management

## Abstract

**Background:**

The aim of this study is to evaluate both the utility of MRI scans and reports used in the current practice routine of shoulder surgeons and their surgical decision-making process.

**Methods:**

Ninety-three shoulder-specialised orthopaedic surgeons of the Canadian Shoulder and Elbow Society (CSES) Orthopaedic Association were surveyed in 2020 anonymously online to help identify the use of MR-imaging and reports in managing shoulder disorders and surgical decision process.

**Results:**

Thirty out of 93 (32.25%) CSES fellowship-trained orthopaedic surgeons participated. Respondents request MRI scans in about 55% of rotator cuff (RC) pathology and 48% of shoulder instability cases. Fifty percent of patients with potential RC pathology arrive with a completed MRI scan prior first orthopaedic consult. Their surgical decision is primarily based on patient history (45–55%) and physical examination (23–42%) followed by MRI scan review (2.6–18%), reading MRI reports (0–1.6%) or viewing other imaging (3–23%) depending on the shoulder disease. Ninety percent of surgeons would not decide on surgery in ambiguous cases unless the MR-images were personally reviewed. Respondents stated that shoulder MRI scans are ordered too frequently prior specialist visit as identified in more than 50% of cases depending on pathology.

**Conclusions:**

The decision-making process for shoulder surgery depends on the underlying pathology and patient history. The results demonstrate that orthopaedic surgeons are comfortable reviewing shoulder MRI scans without necessarily reading the MRI report prior to a surgical decision. MRI scans are becoming an increasingly important part of surgical management in shoulder pathologies but should not be used without assessment of patient history and or physical examination.

## Background

Surgical decision making in shoulder surgery is classically formulated following a patient history, focused on clinical examination supported by (radiologic) imaging. The use of imaging modalities (eg. radiographs, ultrasound, CT, MRI) has changed in the past decade [1].

Particularly, the use of MR-imaging in shoulder disorders has increased significantly in the last decade [1]. MRI scans have revolutionized the management of soft-tissue disorders of the shoulder and other body regions, which were previously difficult to diagnose often requiring user dependent tools such as ultrasound [2].

MR-images are routinely used and read separately by the ordering orthopaedic surgeons and radiologists, the latter specialty generating an MRI report [3, 4]. An additional cause for increased MRI utilization can be the patient generated request or the result of medical legal considerations, often encountered in the Canadian tort system. Patients often ask for MRI scans from their treating doctor, despite of having no indication for the study. They receive the referral often in order to prevent a legal action and for the sake of enhancing the patient-doctor relationship [5, 6]. Despite increase in MR-imaging quality, signals detected may represent false positive pathologies particularly in cases of rotator cuff tears [4, 7, 8]. Changes in signal sensitivity, pixels and the width of the generated slice can lead to a discrepancy between pathologic and non-pathologic images [9, 10]. Normal aging also poses its own challenges particularly as it pertains to asymptomatic rotator cuff tears [11–13]. Therefore, interpreting images can be very difficult, when solely looking at the MRI scans and not being able to enjoy the benefit of a full patient history and physical examination. The purpose of the study was to identify the utility of MRI scans and reports used in the current practice routine of shoulder surgeons and their surgical decision-making process.

## Methods

A web-based survey was developed targeting active sub-specialized orthopaedic surgeons of the Canadian Shoulder and Elbow Society (CSES) which is part of the Canadian Orthopaedic Association. The survey consisted of questions related to the use of MR-imaging and reports in the management of shoulder disorders and the surgical decision process of orthopaedic surgeons.

A web-based survey was developed and administered using Qualtrics (Qualtrics, Provo, UT, USA), an easy-to-use online survey tool and analysis platform. The survey data is kept secure and is stored and backed up in Canada in order to comply with the British Columbia Freedom of Information and Protection of Privacy Act (FIPPA). Ethics approval was obtained for the study from the University of British Columbia research ethics board (CREB H20-01,321). Informed Consent to participate and for publication was obtained from each participant. The study was carried in accordance with the latest Declaration of Helsinki.

Ninety-three CSES orthopaedic surgeons were sent an e-mail with the invitation link to take the survey by the CSES secretary. The survey was distributed at the start of August 2020 and closed end of September 2020.

Thirty orthopaedic surgeons completed the survey and were included in the analysis.

All data was collected anonymously. The results are reported in tabulated form. Descriptive statistics included mean and standard deviation. Categorial variables were reported in sample size and percentiles.

The survey questions and responses are demonstrated in Tables 1, 2, 3 and 4.

**Table 1:** Respondents’ information

**Q1—How old are you?**						
		**%**		**Count**		
< 30 years		0.00%		0		
30–40 years		16.67%		5		
> 40–50 years		46.67%		14		
> 50–60 years		23.33%		7		
> 60–70 years		13.33%		4		
> 70 years		0.00%		0		
Total		100%		30		
**Q2—How would you describe your gender?—Selected Choice**						
		**%**		**Count**		
Male		83.33%		25		
Female		16.67%		5		
Transgender		0.00%		0		
Other ____________ (non-binary, gender-fluid, agender, please specify)		0.00%		0		
Prefer not to say		0.00%		0		
Total		100%		30		
**Q3—Since how many years are you a fully qualified orthopaedic surgeon?**						
	**Minimum**	**Maximum**	**Mean**	**Std Deviation**	**Variance**	**Count**
	5	35	17.6	8.91	79.37	30
**Q4—Have you completed fellowship training?**						
		**%**		**Count**		
Yes		100.00%		30		
No		0.00%		0		
Total		100%		30		
**Q5—What is your sub-specialty? – Selected Choice**						
		**%**		**Count**		
Shoulder surgeon		33.33%		10		
Upper Limb surgeon		36.67%		11		
Sports arthroscopy		26.67%		8		
Other		3.33%		1		
Total		100%		30		
**Q6—How many shoulder specific patients do you see on average per month?**						
		**%**		**Count**		
< 50		6.67%		2		
50—100		30.00%		9		
> 100—200		23.33%		7		
> 200		40.00%		12		
Total		100%		30

**Table 2:** Patients and imaging

**Q7—What percentage of … cases do arrive at your consultation office already with a completed MRI scan?**						
	**Mean**	**Minimum**	**Maximum**	**Std Deviation**	**Variance**	**Count**
a) Rotator cuff pathology (including calcific tendinitis and biceps pathology)?	50.71	10	100	25.49	649.49	28
b) Shoulder instability?	33.57	0	80	21.58	465.82	28
c) Frozen shoulder?	25.56	0	80	22.33	498.77	27
d) Glenohumeral arthritis?	26.15	0	70	18.41	339.05	26
e) ACJ-Pathology?	24.8	0	80	23	528.96	25
**Q8—What percentage of … cases do you order an MRI scan?**						
	**Mean**	**Minimum**	**Maximum**	**Std Deviation**	**Variance**	**Count**
a) Rotator cuff pathology (including calcific tendinitis and biceps pathology)?	55.36	10	100	29.7	882.02	28
b) Shoulder instability?	48.15	0	100	33	1089.16	27
c) Frozen shoulder?	11.5	0	80	19.31	372.75	20
d) Glenohumeral arthritis?	20	0	80	25.3	640	20
e) ACJ-Pathology?	17	0	80	23.26	541	20
**Q9—What percentage of … cases do you get a CT scan instead?**						
	**Mean**	**Minimum**	**Maximum**	**Std Deviation**	**Variance**	**Count**
a) Rotator cuff pathology (including calcific tendinitis and biceps pathology)?	2	0	10	4	16	15
b) Shoulder instability?	41.85	0	100	31.04	963.24	27
c) Frozen shoulder?	3.13	0	40	9.82	96.48	16
d) Glenohumeral arthritis?	66.43	0	100	34.87	1215.82	28
e) ACJ-Pathology?	2.14	0	20	5.58	31.12	14
**Q10—What percentage of … cases do you get an Ultrasound instead?**						
	**Mean**	**Minimum**	**Maximum**	**Std Deviation**	**Variance**	**Count**
a) Rotator cuff pathology (including calcific tendinitis and biceps pathology)?	33.6	0	100	30.84	951.04	25
b) Shoulder instability?	1.43	0	10	3.5	12.24	14
c) Frozen shoulder?	13.33	0	50	19.55	382.22	15
d) Glenohumeral arthritis?	17.33	0	90	28.63	819.56	15
e) ACJ-Pathology?	6	0	50	13.06	170.67	15
**Q11—Do you personally look at the MRI images in cases of … ?**						
	Yes		No		Total	
a) Rotator cuff pathology (including calcific tendinitis and biceps pathology)?	96.55%	28	3.45%	1	29	
b) Shoulder instability?	96.55%	28	3.45%	1	29	
c) Frozen shoulder?	89.66%	26	10.34%	3	29	
d) Glenohumeral arthritis?	93.10%	27	6.90%	2	29	
e) ACJ-Pathology?	89.66%	26	10.34%	3	29	
**Q12—Do you read the MRI report in cases of … ?**						
	Yes		No		Total	
a) Rotator cuff pathology (including calcific tendinitis and biceps pathology)?	89.66%	26	10.34%	3	29	
b) Shoulder instability?	89.66%	26	10.34%	3	29	
c) Frozen shoulder?	89.66%	26	10.34%	3	29	
d) Glenohumeral arthritis?	79.31%	23	20.69%	6	29	
e) ACJ-Pathology?	79.31%	23	20.69%	6	29	
	**Q13—If No, why do you not read the report?**					
	Answer	%	Count			
	often false positive	42.86%	3			
	often false negative	0.00%	0			
	takes too much time	0.00%	0			
	often not at hand	14.29%	1			
	other	42.86%	3			
	Total	100%	7			
	**Q14—If Yes, why do you read the report?**					
	Answer	%	Count			
	do not want to miss something	15.38%	4			
	to double-check one’s personal diagnosis	61.54%	16			
	because it helps my surgical decision	3.85%	1			
	other	19.23%	5			
	Total	100%	26

**Table 3:** Surgical decision process

**Q15—What makes you generally decide on surgery in cases of …? Rotator cuff pathology (including calcific tendinitis & biceps pathology)**						
	**Mean**	**Minimum**	**Maximum**	**Std Deviation**	**Variance**	**Count**
Patient history	48.15	29	81	15.42	237.9	27
Physical exam	29.85	9	49	9.21	84.79	27
MRI images	18.04	0	49	12.25	150.18	27
MRI report	0.89	0	9	2.44	5.95	27
Other imaging (ultrasound, x-ray, CT)	3.07	0	21	5.08	25.85	27
**Q16—What makes you generally decide on surgery in cases of …? Shoulder instability**						
	**Mean**	**Minimum**	**Maximum**	**Std Deviation**	**Variance**	**Count**
Patient history	55.3	10	100	19.42	377.32	27
Physical exam	28.56	0	90	18.62	346.54	27
MRI images	6.48	0	27	8.86	78.55	27
MRI report	1.59	0	35	6.72	45.2	27
Other imaging (ultrasound, x-ray, CT)	8.07	0	35	10.92	119.18	27
**Q17—What makes you generally decide on surgery in cases of …? Frozen shoulder**						
	**Mean**	**Minimum**	**Maximum**	**Std Deviation**	**Variance**	**Count**
Patient history	48.67	19	100	18.4	338.67	27
Physical exam	42.19	0	80	16.91	285.93	27
MRI images	3.63	0	29	7.55	56.97	27
MRI report	1	0	27	5.1	26	27
Other imaging (ultrasound, x-ray, CT)	4.52	0	33	8.4	70.62	27
**Q18—What makes you generally decide on surgery in cases of …? Glenohumeral arthritis**						
	**Mean**	**Minimum**	**Maximum**	**Std Deviation**	**Variance**	**Count**
Patient history	51.22	20	100	21.31	454.02	27
Physical exam	23	0	46	11.78	138.74	27
MRI images	2.63	0	31	7.24	52.46	27
MRI report	0	0	0	0	0	27
Other imaging (ultrasound, x-ray, CT)	23.15	0	72	20.34	413.61	27
**Q19—What makes you generally decide on surgery in cases of …? ACJ-Pathology**						
	**Mean**	**Minimum**	**Maximum**	**Std Deviation**	**Variance**	**Count**
Patient history	45.3	24	100	18.06	326.06	27
Physical exam	37.19	0	71	14.64	214.37	27
MRI images	3.59	0	35	8.27	68.46	27
MRI report	0.33	0	9	1.7	2.89	27
Other imaging (ultrasound, x-ray, CT)	13.59	0	35	12.32	151.87	27
**Q20—After taking patient history and physical examination, how would you prioritize surgical decision making in cases of Rotator cuff pathology (including calcific tendinitis & biceps pathology) by …**						
	Mean	Minimum	Maximum	Std Deviation	Variance	Count
reading the MRI images yourself	56.56	0	100	39.11	1529.43	27
reading the MRI report alone?	3	0	20	5.62	31.63	27
reading both MRI images + report?	51.19	0	100	38.17	1456.82	27
**Q21—After taking patient history and physical examination, how would you prioritize surgical decision making in cases of Shoulder instability by …**						
	Mean	Minimum	Maximum	Std Deviation	Variance	Count
reading the MRI images yourself	58	0	100	38.61	1490.67	27
reading the MRI report alone?	2.59	0	44	8.82	77.8	27
reading both MRI images + report?	46.67	0	100	39.17	1534.52	27
**Q22—After taking patient history and physical examination, how would you prioritize surgical decision making in cases of Frozen shoulder by …**						
	Mean	Minimum	Maximum	Std Deviation	Variance	Count
reading the MRI images yourself	43.63	0	100	43.51	1893.49	27
reading the MRI report alone?	2.15	0	26	5.81	33.76	27
reading both MRI images + report?	46	0	100	41.51	1723.04	27
**Q23—After taking patient history and physical examination, how would you prioritize surgical decision making in cases of Glenohumeral arthritis by**						
	Mean	Minimum	Maximum	Std Deviation	Variance	Count
reading the MRI images yourself	41.56	0	100	43.67	1907.14	27
reading the MRI report alone?	0.3	0	5	1.08	1.17	27
reading both MRI images + report?	39.74	0	100	43.7	1910.12	27
**Q24—After taking patient history and physical examination, how would you prioritize surgical decision making in cases of ACJ-Pathology by**						
	Mean	Minimum	Maximum	Std Deviation	Variance	Count
reading the MRI images yourself	45.96	0	100	45.28	2050.33	27
reading the MRI report alone?	1.41	0	17	3.95	15.57	27
reading both MRI images + report?	42.93	0	100	43.27	1872.22	27

**Table 4:** MRI use and diagnosis

**Q25 Would you solely decide for surgery based on the MRI report in an ambiguous patient history or examination (without seeing the actual images) in cases of …?**																
					Yes			No				Total				
a. Rotator cuff pathology (including calcific tendinitis & biceps pathology)?					7.41%		2	92.59%		25		27				
b. Shoulder instability?					7.41%		2	92.59%		25		27				
c. Frozen shoulder?					7.41%		2	92.59%		25		27				
d. Glenohumeral arthritis?					11.11%		3	88.89%		24		27				
e. ACJ-Pathology?					7.41%		2	92.59%		25		27				
**Q26—What are your concerns regarding indications/situations of MRI scans (images and reports) and reliability in cases of …? (up to 3 answers per disorder possible)**																
	Done too fast/early		False positivity		Misinterpretation by patient			Not ordered by the specialists				Discrepancy between clinical examination and MRI images & reports			Total	
a. Rotator cuff pathology (including calcific tendinitis & biceps pathology)?	9.68%	6	16.13%	10	30.65%		19	14.52%			9	29.03%		18	62	
b. Shoulder instability?	20.75%	11	5.66%	3	22.64%		12	16.98%			9	33.96%		18	53	
c. Frozen shoulder?	16.67%	9	14.81%	8	22.22%		12	16.67%			9	29.63%		16	54	
d. Glenohumeral arthritis?	9.80%	5	9.80%	5	25.49%		13	29.41%			15	25.49%		13	51	
e. ACJ-Pathology?	14.00%	7	18.00%	9	22.00%		11	18.00%			9	28.00%		14	50	
**Q27—What is in your opinion the best indication/situation in regards to MRI scans (images and reports) helping with the diagnosis and reliability in orthopaedic shoulder surgery?**																
				Minimum		Maximum		Mean	Std Deviation		Variance		Count			
Rotator cuff pathology?—in Full thickness tears				0		100		67.69	30.42		925.44		26			
Rotator cuff pathology?—in Partial thickness tears				0		100		61.6	32.7		1069.44		25			
Rotator cuff pathology?—in Massive rotator cuff tears				0		100		74.44	25.72		661.73		27			
Rotator cuff pathology?—in Calcified tendinitis				0		80		31.67	25.44		647.22		24			
Rotator cuff pathology?—in SLAP-Lesions				0		100		47.39	32.06		1027.98		23			
Rotator cuff pathology?—in LHB-Tendon tears				0		100		38.64	25.99		675.41		22			
Shoulder instability				0		100		55.22	31.47		990.17		2 3			
Frozen shoulder				0		50		13.89	13.8		190.43		18			
Glenohumeral arthritis				0		80		23.16	24.07		579.5		19			
ACJ-Pathology				0		50		20.5	18.83		354.75		20			
**Q28—Have you ever used an MRI scan in order to help convince a patient to postpone patients’ need for surgery in cases of …?**																
						Yes			No				Total			
a. Rotator cuff pathology (including calcific tendinitis & biceps pathology)?						57.69%		15	42.31%		11		26			
b. Shoulder instability?						26.92%		7	73.08%		19		26			
c. Frozen shoulder?						34.62%		9	65.38 %		17		26			
d. Glenohumeral arthritis?						11.54%		3	88.46%		23		26			
e. ACJ-Pathology?						15.38%		4	84.62%		22		26			
**Q29—Do you think MRI scans are been over-used in … ?**																
						Yes			No				Total			
a. Rotator cuff pathology (including calcific tendinitis & biceps pathology)?						53.85%		14	46.15%		12		26			
b. Shoulder instability?						73.08%		19	26.92%		7		26			
c. Frozen shoulder?						88.46%		23	11.54%		3		26			
d. Glenohumeral arthritis?						88.46%		23	11.54%		3		26			
e. ACJ-Pathology?						92.31%		24	7.69%		2		26			
**Q30—How often do you require a radiologist to review the MRI with you before you make a decision on surgery in cases of …?**																
						Minimum		Maximum	Mean		Std Deviation		Variance			Count
a) Rotator cuff pathology (including calcific tendinitis and biceps pathology)?						0		20	4.62		6.34		40.24			26
b) Shoulder instability?						0		30	6.92		8.67		75.15			26
c) Frozen shoulder?						0		10	2.69		4.44		19.67			26
d) Glenohumeral arthritis?						0		20	3.46		6.17		38.02			26
e) ACJ-Pathology?						0		20	3.08		6.06		36.69			26
**Q31—For all diagnosis, what percentage of cases would you feel comfortable making a decision without any radiologist input (including report) in cases of …?**																
						Minimum		Maximum	Mean		Std Deviation		Variance			Count
a) Rotator cuff pathology (including calcific tendinitis and biceps pathology)?						0		100	84.62		22.05		486.39			26
b) Shoulder instability?						0		100	82.31		27.92		779.29			26
c) Frozen shoulder?						0		100	86.15		24.82		615.98			26
d) Glenohumeral arthritis?						0		100	91.15		21.54		464.05			26
e) ACJ-Pathology?						0		100	93.08		21.08		444.38			26

## Results

Thirty out of 93 (30%) active CSES fellowship-trained orthopaedic surgeons participated in the online survey. Most respondents are 40 – 60 years old (*n* = 21; 70%) and male (*n* = 25; 83.3%) (Table 1). All of the survey respondents had completed at least one year of fellowship training (1–2 years: *n* = 28; 93.3%) and have been practicing on average 17.6 ± 8.9 years. The respondents are predominantly upper limb (*n* = 11; 36.7%) and shoulder surgeons (*n* = 10; 33.3%). They see clinically between 50 and more than 200 shoulder specific patients per month (*n* = 28; 93.3%).

Surgeons report that 50.7 ± 25.5% of their patients arrive for their first visit with completed MRI scans in cases of potential rotator cuff pathology (Table 2). In other diseases such as shoulder instability, frozen shoulder, glenohumeral osteoarthritis (GHOA) or acromioclavicular joint (ACJ) pathology, the rate of pre-assessment MRI falls to between 25 – 33.5%. The respondents order MRI scans when suspecting rotator cuff pathology (RCP) and shoulder instability in 55.4% and 48.2% of cases, respectively. Ultrasound scans are favoured as an adjunct for RCP (33.6 ± 30.1%). Computer tomography (CT) scans are uncommonly ordered for RCP (2 ± 4%), frozen shoulder (3.3 ± 9.8%) or ACJ pathology (2.1 ± 5.6%), but CT scans are used extensively for preoperative planning in cases of GHOA (66.4%) and shoulder instability (41.9%).

Most surgeons (90–97%) review the MRI scans personally, and less review the MRI report (80–90%) (Table 2). The main reason (85%) for not reading the MRI report is the concern of false positive/negative reports. Reasons cited for reading the MRI report include the need to confirm one’s personal diagnosis (65.4%) and to avoid missing comorbid pathology (23.1%).

When looking at five factors influencing surgical decision making (patient history, physical examination, MRI images, MRI report and other imaging modalities), the most important factor remains patient history (depending on pathology 45.3 – 55.3%) followed by physical examination (pathology dependent 23 – 42.2%) (Table 3). CSES surgeons rely on MRI images mainly in cases of RCP (18.4 ± 12.3%) for surgical decision making. MRI reports alone are rarely used in various shoulder pathologies (0 – 1.6%). Other imaging modalities (eg. CT) are primarily used in cases of GHOA (23.2 ± 20.3%) and ACJ pathology (13.6 ± 12.3%).

Once patient history and physical examination are completed, the upper extremity surgeon reads the MRI images alone or in combination with the MRI report as demonstrated in Table 3. The percentage of surgeons who review the MR images in isolation depends on the shoulder disorder. The main disorders are shoulder instability (58 ± 38.6%) and RCP (56.6 ± 39.1%) (Table 3). The MRI report alone is used sparingly reaching its maximum with 3% in RCP and its minimum with 0.3% in cases of GH-OA.

Data out of Table 4 demonstrates that approximately 90% (88.9–92.6%) of respondents would not make a surgical decision in ambiguous cases of shoulder disorders without seeing the actual MRI images. Surgeons report concerns regarding MRI images and reports as they often perceive a discrepancy between the clinical examination findings and the MRI report (25.5 – 34.0%), and furthermore they note that this difference can cause a misconception of proper diagnosis by the patient in 22 – 30.6% of cases which can and will alter patient expectations. The propensity of the above is dependent on the type of shoulder pathology. The respondents designate that the best indication for MRI scans in order to evaluate and plan surgical management is in cases with RCP with massive cuff tears (74.4 ± 25.7%), full thickness tears (67.7 ± 30.4%), or partial thickness tears (61.6 ± 32.7%), or cases of shoulder instability (55.2%), which is interesting given the proclivity of bone loss affecting surgical choice (e.g. Bankart versus Latarjet procedures).

MRI scans have been used by some surgeons to postpone or counsel against a surgery particularly in cases of RCP (57.7%) and frozen shoulder (34.6%) (Table 4). Further, respondents advise strongly that MRI scans are over-ordered particularly in cases of frozen shoulder (88.5%), GHOA (88.5%) and ACJ pathology (92.3%). Very few surgeons feel the need to review the MRI images with a radiologist given their subspecialty training prior to surgery (depending on shoulder pathology 2.7 – 6.9%). The majority of CSES surgeons report that they feel comfortable reviewing shoulder MRI scans and making surgical decisions without the help input of a radiologist (82.3 – 93.1%).

## Discussion

The survey results demonstrate that orthopaedic sub-specialty shoulder surgeons from the CSES primarily believe that MRI scans should be requested in suspected cases of rotator cuff pathology and shoulder instability, but only after a comprehensive patient history and physical examination. Furthermore, the results demonstrate that an excessive number of patients particularly with suspected rotator cuff pathology (approximately 50%); already arrive with a completed MRI scan prior to consultation with the orthopaedic specialist. The MRI report is useful mainly in combination with personal review of MR images to advise for and against shoulder surgery. Ninety percent of respondents would not make a surgical decision solely on an MRI report.

Shoulder disorders can present in a variety of pain patterns and the establishment of the correct diagnosis can be difficult due to a multitude of pain generators and various combinations of pathologies. Modern technological advancements in radiologic imaging have helped surgeons clinch the diagnosis more rapidly and less invasively [1]. Recently, the overall use of MRI scans has increased drastically [14, 15]. Fifty percent of patients, arrive for consultation with a completed shoulder MRI scan. The non-specialist ordered scans are supposed to speed up the diagnostic and surgical decision-making process. Paradoxically surgery is not indicated in 66% (*n* = 182 of 275) of patients with ordered MRI scans, and 71.3% (*n* = 196 of 275) of those MRI studies were already pre-ordered and completed before presenting to the shoulder and elbow specialists, as described by Reynolds et al. [16]. In Canada, patients are initially seen by their general practitioner who manages them non-operatively (e.g. pain medications, prescribes physiotherapy), or refers them to sports medicine doctors for additional non-surgical treatments, or to orthopaedic surgeons for surgical management [17]. If the patient has been treated for an extended time period or in order to help expedite the diagnosis and treatment, patients might have already obtained radiologic imaging before arriving at the orthopaedic surgeon.

Furthermore, there is an argument to be made that MR scans should not be ordered by generalists. Properly indicated MRI scans could reduce the chance of potential collateral medical exposure risks to 15% of patients, free up capacity for urgently needed MRI scans and cut costs by more than US$ 26,000 over 12 months [16]. Another reason to reduce aggressive testing/MRI scanning is the limited ability to glean useful information in certain diagnoses [18]. Furthermore, freeing up MRI scanners for more urgent cases is important as the availability of MRI scans is limited in some countries more than in others [19].

Nonetheless, respondents demonstrated that appropriately ordered MRI scans are important and they also order MRI’s frequently in cases of rotator cuff pathology or shoulder instability. Surgeons personally review the MR-images in 90–97% of cases depending on pathology, and surgeons are less likely to read the MRI report (80–90% of times). The MRI report is usually read after the viewing the MR-images. Finally, the MRI report is very seldomly reviewed in isolation (0.3–3%). This is similar to the described survey results by Kruger et al. on orthopaedic surgeons reading radiology reports in addition to viewing images for any imaging modality [20].

Generally, MRI reports are still among the most read reports with 92% and this percentage decreases dramatically for reports involving other modalities such as ultrasound (74%), CT scan (39%) or plain radiography (10%) [20]. Kruger et al. stated that 55% of orthopaedic surgeons would disagree with the written MRI report according to their survey data [20]. The current study illustrates that 25.5 – 34.0% of misinterpretation exists between the personally reviewed MRI shoulder images and the generated radiology report. Accordingly, less than five percent of these respondents felt comfortable reading MRI reports without viewing the images by themselves. The main reason cited (85%) for not reading the MRI report is that the generated reports often conveyed false positive information (5.6 – 18%) confounding the provisional diagnosis. However, one has to keep in mind that the orthopaedic surgeon has an advantage compared to the radiologist, because the orthopaedic surgeon is able to review the images, following completion of patient history and performance a physical examination. The latter two are the most important assessment means in establishing the diagnosis and decision on surgical treatment of shoulder disorders as identified in the questionnaire. This allows a more discerning “eye” interpreting the MR images. Another very important factor is the level of training of the radiologists. Radiologists with a musculoskeletal fellowship will feel more comfortable reviewing MRI scans of the shoulder than an interventional radiologist or one with gastrointestinal fellowship training [21]. This begs the question of whether MRI interpretation is valid without the advantage of a pre-study informed history and physical examination or specific level of training. According to the respondents, reviewing the MR-images by themselves or with the report is favoured by the majority. This is congruent with the results of demonstrating the respondents feeling comfortable reviewing the MRI scans independently. However, the MRI report/the radiologist interprets the MR-images in its’ entirety and hence can detect other/non-musculoskeletal pathologies [22], which often are missed by the orthopaedic surgeon due to a focused analysis. Thus, reviewing the MRI report can help identify any additional abnormalities in other non-focused areas.

The study has limitations. It is a survey targeting a specific complicated body region and has a very limited number of respondents. However, the online survey participation rate of 32.25% is in the normal participation range for surveys [23]. There was no separation into traditional MRI scans and MR-arthrograms. Nonetheless, an MR-arthrogram of the shoulder has a higher sensitivity (78%) and specificity (100%) when compared to a regular MRI, 72% and 78% respectively [24]. Further, the MR-arthrogram is mostly ordered with a very specific question that can be much better addressed in the MR-arthrogram than in the regular MRI leading to a more precise report by the radiologist e.g. labral pathology. Additionally, the request for an MR-arthrogram is generally ordered and sometimes limited to the request of an orthopaedic surgeon with a very detailed question regarding soft-tissues pathologies. Thus, the false-positive ratio of potential pathologies in the report is decreased [24, 25]. Furthermore, the expert opinion of highly training shoulder surgeons demonstrates the trouble MRI scans can cause for patients confounding the diagnosis and confusing treatment/surgical management process. This report quantifies both shoulder surgeon read images versus merely reading the report plus reasons for their preconception as it pertains to decision making for shoulder surgery.

## Conclusions

Surgical decision making varies according to underlying shoulder pathology and patient history. The results demonstrate that orthopaedic surgeons are comfortable reviewing shoulder MRI scans without necessarily reading the MRI report prior to a surgical decision. Furthermore, more than half of the respondents feel that MR-imaging is too frequently used for non-rotator cuff pathology. Finally, MRI scans are an increasingly important part of surgical management in shoulder pathologies but should not be used without assessment of patient history and or physical examination. Finally, there is little to no emphasis placed on reading radiology reports of the same scans.

## Data Availability

All data generated or analysed during this study are included in this published article.
